# Microbiota and neurologic diseases: potential effects of probiotics

**DOI:** 10.1186/s12967-016-1058-7

**Published:** 2016-10-19

**Authors:** Giulia Umbrello, Susanna Esposito

**Affiliations:** Pediatric Highly Intensive Care Unit, Department of Pathophysiology and Transplantation, Università degli Studi di Milano, Fondazione IRCCS Ca’ Granda Ospedale Maggiore Policlinico, Via Commenda 9, 20122 Milan, Italy

**Keywords:** Autism spectrum disorder, Brain, Gut microbiota, Microbiota, Probiotics

## Abstract

**Background:**

The microbiota colonizing the gastrointestinal tract have been associated with both gastrointestinal and extra-gastrointestinal diseases. In recent years, considerable interest has been devoted to their role in the development of neurologic diseases, as many studies have described bidirectional communication between the central nervous system and the gut, the so-called “microbiota-gut-brain axis”. Considering the ability of probiotics (i.e., live non-pathogenic microorganisms) to restore the normal microbial population and produce benefits for the host, their potential effects have been investigated in the context of neurologic diseases. The main aims of this review are to analyse the relationship between the gut microbiota and brain disorders and to evaluate the current evidence for the use of probiotics in the treatment and prevention of neurologic conditions.

**Discussion:**

Overall, trials involving animal models and adults have reported encouraging results, suggesting that the administration of probiotic strains may exert some prophylactic and therapeutic effects in a wide range of neurologic conditions. Studies involving children have mainly focused on autism spectrum disorder and have shown that probiotics seem to improve neuro behavioural symptoms. However, the available data are incomplete and far from conclusive.

**Conclusions:**

The potential usefulness of probiotics in preventing or treating neurologic diseases is becoming a topic of great interest. However, deeper studies are needed to understand which formulation, dosage and timing might represent the optimal regimen for each specific neurologic disease and what populations can benefit. Moreover, future trials should also consider the tolerability and safety of probiotics in patients with neurologic diseases.

## Background

In recent years, the gut microbiota residing in the gastrointestinal tract have emerged as a topic of great interest in medical research. The gut microbiota consist of trillions of microorganisms representing many different species of known bacteria, as well as viruses, fungi, *protozoa* and *archaea* [1, 2]. Among the various bacteria, the most abundant phyla are *Bacteroidetes* and *Firmicutes*, followed by *Proteobacteria* and *Actinobacteria*, while *Fusobacteria* and *Verrucomicrobia* are less common. Butyrate-producing bacteria and lactic acid bacteria are thought to have beneficial effects to the host through anti-inflammatory, anti-tumourigenic and pathogen-exclusion properties [3].

The deep influence of the gut microbiota on human health and homeostasis has many clinical manifestations. Studies have shown how dysbiosis (i.e., a disruption of the balanced composition of the gut microbiota) is associated with gastrointestinal [4–7] and extra-gastrointestinal diseases [8–10]. Moreover, recent investigations have also advocated a possible role for microbiota in the pathogenesis of several brain disorders [11–13]. The emerging idea of the microbiota as a modulator of neural physiology has recently been investigated through the concept of the “microbiota-gut-brain axis”, which represents a composite model of interaction between the intestinal microbes and the brain. Despite the evidence of such communication, the effect, magnitude and clinical relevance of the disruption of the microbiota in neurologic diseases have yet to be clearly elucidated.

In view of these considerations, the potential role of probiotics in the prevention and treatment of neurologic diseases presents an attractive possibility. Probiotics are living non-pathogenic microorganisms that confer a health benefit and improve physiological conditions in the host when administered in adequate amounts, as a food ingredient, supplement or drug [14]. Probiotics are mainly composed of lactic-acid bacteria, such as *Lactobacilli, Lactococci* and *Bifidobacteria* or yeasts as *Saccharomycetes*; to date, *Lactobacillus rhamnosus GG, Lactobacillus casei, Lactobacillus plantarum, Lactobacillus johnsonii, Bifidobacterium* and *Saccharomyces boulardii* are the most widely studied strains [15].

Although the exact mode of action of probiotics remains uncertain, it is likely that several mechanisms operate together. Probiotics exert a microbiological function by preventing opportunistic pathogens from occupying functional niches in the gut microbial community, blocking epithelial attachment of pathogenic bacteria, inhibiting their growth with the production of lactic acid, propionic acid, acetic acid, bacteriocins and reactive oxygen species [16–19]; they also play a nutritional role by producing several vitamins, lactase and health-promoting compounds [16–19]. In addition, they regulate intestinal transit and reinforce the gut barrier. Furthermore, probiotics play an important role in the regulation of both the innate and adaptive immune systems by activating macrophages, NK cells and cytotoxic T cells, modulating the production of IgA, stimulating toll-like receptors and modifying the cytokine-expression profile [16–19].

Some differences in biological properties and clinical effects have been reported among the various probiotic strains, mainly caused by genetic diversity and host-bacteria interactions [20]. A great deal of evidence has established that the effects of probiotics may be genus-specific and even species- or strain-dependent [21–23] and that their efficacy is influenced by the dose [24]. Moreover, there are differences even between single- and multi-strain probiotic formulations; however, it is not clear whether supplementation with mixtures is better than using a single strain. On examination of 16 comparative studies, probiotic combinations appeared to be more effective than single components taken alone in 12 cases (75 %) [25], although in many studies, this comparison is biased because of differences in dose. It is possible that the presence of a wide variety of probiotic genera in a multi-strain preparation leads to lower efficacy because of mutual inhibition by different species. However, there are data supporting the idea that mixtures have superior effectiveness over single strains, possibly because of a greater concentration of probiotics, a broader range of action and synergistic effects [26].

Probiotics are used as an adjuvant therapy for many paediatric gastrointestinal and extra-intestinal diseases, but few data are available on their use in brain disorders. The main aims of this review are to analyse the relationship between the gut microbiota and brain disorders and to evaluate the current evidence for the use of probiotics in neurologic conditions. Particular attention is paid to factors that condition the modification of the gut microbiota and the possibility of managing neurological diseases by modifying the gut microbial composition. PubMed was used to search for all of the studies published over the last 15 years using the key word “microbiota” and “gut” or “intestinal” and “nervous system”. More than 350 articles were found, and only those published in English and providing data on aspects related to neurologic diseases were included in the evaluation.

## Discussion

### The microbiota-gut-brain axis

Accumulating evidence has shown that gut microbiota influence human brain development and function [27–30]. The exchange of regulatory signals through an integrative, bidirectional communication between the gastrointestinal tract and the central nervous system represents the gut-brain axis [31, 32]. In this relationship, the gut microbiota play a pivotal role. This complex system acts via direct and indirect mechanisms that involve neural, hormonal and immunological pathways [33–35].

In top-down signalling, the central nervous system influences the gut microbiota, mainly through the autonomic nervous system and the hypothalamus–pituitary–adrenal axis. Indeed, several studies have demonstrated that a stressful event, especially early in life, can disrupt the microbiota profile, limit its richness and diversity, and affect bacterial species [36–40], inducing a shift in microbial composition that may promote the translocation of species known to induce inflammation, such as *Clostridia*, and reduce the proportion of anti-inflammatory bacteria, such as *Lactobacillus*.

Another pathway that can produce effects on the brain and behaviour involves the vagus nerve [41]. The modulation of the gut microbiota using *Lactobacillus rhamnosus* stimulated the transcription of γ-aminobutyric acid (GABA) receptors and induced behavioural and psychological responses with marked dependence on vagal integrity [42]. However, vagus-independent mechanisms are involved as well [33]. The intestinal microbiota has a profound influence on several neurotransmitters and neuromodulators, such as monoamines, serotonin, GABA, and brain-derived neurotrophic factor [43–46], which deliver signals to the brain trough enteric nerves, enterochromaffin cells [27] and the systemic circulation, crossing the blood–brain barrier [34], whose permeability appears to be regulated by the microbiota in experimental models [47].

Moreover, the gut microbiome induces maturation of the host immune system, contributes to establishing a durable immune repertoire and modulates the innate and adaptive immune systems to support the dominance of regulatory networks that prevent inflammation or immune-mediated disease and inflammatory responses [48, 49]. In addition, microbiota protect the intestinal barrier by improving epithelial tight junctions, thus reducing gut permeability; in fact, a damaged intestinal wall can lead to increased translocation of gut bacteria into the mesenteric lymphoid tissue, provoking immune and inflammatory responses and activating the vagus nerve and spinal afferent neurons. Inflammatory cytokines and the vagal system, in turn, affect the activity of the central nervous system, altering its function [50]. Interestingly, recent data reported the activation of inflammasomes—systems involved in the regulation of inflammatory responses through the production of proinflammatory cytokines in children with ASD, suggesting a possible new link between impaired gastrointestinal permeability and neuroinflammation [51].

Neural pathways originate in utero and continue to develop in early postnatal life [52]. It has been shown that the prenatal and postnatal early-life periods are both dynamic and vulnerable windows for brain development. During these important neurodevelopmental phases, essential processes and structures are established. Exposure to adverse events that interfere with this critical sequence of events confers a high risk for the subsequent emergence of mental illness later in life [52]. It is increasingly accepted that the gastrointestinal microbiota contributes substantially to shaping the development of the central nervous system. Conversely, several studies have shown that early-life events can also impact on this gut community [52]. Due to the bidirectional communication between the gut and the brain, it is possible that aberrant situations affecting either organ in early life can impact on the other. Studies have now shown that deviations from the gold standard trajectory of gut microbiota establishment and development in early life can lead not only to disorders of the gastrointestinal tract but also complex metabolic and immune disorders. Moreover, the gut microbiome, too, undergoes dramatic dynamic changes during growth, especially throughout infancy and childhood [53]. Metagenomic studies suggest that the microbiome may also evolve later, as adolescents have significantly different microbiota from adults [54]. Because maturation of the brain and microbiota appears to be concomitant, occurring in parallel and during similar periods, the existence of a critical window during which microbiota can influence brain development and vice versa has been postulated [55, 56]. Preclinical studies on germ-free (GF) mice demonstrated that abnormalities in neural growth factors and altered behaviours could be normalized by conventional bacterial colonization occurring early in life, supporting the idea of a developmental window for gut-brain interaction, where some effects are especially important in childhood [57]. Furthermore, according to a recent study, the disruption of microbiota with high doses of antibiotics from weaning onwards could alter brain development and behaviour despite the presence of normal gut microbiota in early postnatal life [58]. These data extend the time window during which perturbations of microbiome and dysregulation of gut microbiome-brain axis may influence brain health to adolescence and early adulthood.

Considering these interactions and the emerging role of microbiota as a key element of neural development and regulation, adverse changes in the microbiota may cause alterations in neural networks, affecting general and mental health. A disruption during dynamic periods such as childhood and adolescence could alter brain-gut communication, increasing the risk of neurodevelopmental and other brain disorders later in life [56, 59]. Thus, early pre-weaning and childhood seem to be the critical ages for modulating the intestinal microbiota by avoiding dysbiosis using probiotics to prevent neurologic disorders.

### Microbiome disruption in neurologic diseases

The alteration of microbiota-gut-brain axis interactions has been advocated as a possible cause of some brain diseases, including ASD, Parkinson’s disease (PD), multiple sclerosis (MS) and mood disorders [13, 60–62]. However, there is still little evidence regarding the underlying mechanisms responsible, and there is no consensus on the importance of intestinal dysbiosis in the pathogenesis of neurologic diseases.

Based on pre-clinical evidence, the microbiota have proved essential for the development of experimental autoimmune encephalomyelitis (EAE). GF mice did not develop EAE in a spontaneous model [63] or showed significantly attenuated disease in an inducible model [64]. In addition, the depletion of commensal microflora with oral antibiotics significantly reduced the severity of EAE when compared to control mice treated with intraperitoneal antibiotics or treated with phosphate-buffered saline [65], whereas bacterial colonization of GF mice reestablished EAE susceptibility [63]. Furthermore, in amyotrophic lateral sclerosis transgenic mice, butyrate-producing bacteria (*Butyrivibrio fibrisolvens*), *Escherichia coli*, and *Firmicus* sp. were significantly reduced compared to wild-type mice, providing evidence of dysbiosis [66]; however, it is not known whether the altered microbiome plays an active role in neuromuscular degeneration in amyotrophic lateral sclerosis.

In studies performed on adults, a reduced percentage of *Prevotellaceae* was detected in stool samples of PD patients compared to controls [67], while a greater abundance of *Enterobacteriaceae* was positively associated with the severity of postural instability and gait-difficulty symptoms. Another finding among patients suffering from PD compared to controls was an increased level of urinary indoxyl sulphate unrelated to the presence of constipation, which suggested that the intestinal dysbiosis has already occurred at the onset of the disease and thus possibly plays a role in PD pathogenesis [68]. Human studies have also been performed on patients with MS: despite the limited number of subjects, these studies overall found variations in microbiota bacteria that may play a role in the inflammatory process of the disease [69].

Most of the studies of children concern ASD. Despite agreement among researchers in describing several differences in the composition of the microbiota between ASD children and healthy siblings or unrelated controls [70], the studies sometimes yielded contradictory results about the nature or amount of microbes involved [71].

Finegold et al. studied the faecal microflora of 33 children with ASD using a pyrosequencing technique and found that patients with ASD compared to controls had a higher proportion of *Bacteroidetes* and *Proteobacteria* and a lower abundance of *Firmicutes* and *Actinobacteria* (especially *Bifidobacterium*) [72]. These results are consistent with the results of other studies that reported lower levels of *Bifidobacterium* species in children with ASD [73, 74]. Other abnormalities in composition of the gut microbiota of ASD children included a significantly higher proportion of *Bacteroides vulgatus* [72], *Sutterella, Ruminococcus torques* [75], and *Desulfovibrio* sp. [72, 76]. The latter has been proposed as an important bacterium in the pathophysiology of ASD [77].

Moreover, markedly lower percentages of *Prevotella, Coprococcus* and unclassified *Veillonellaceae* were reported in ASD children with gastrointestinal disorders compared to healthy controls, and these alterations were related to the severity of ASD symptoms [78]. Furthermore, several studies showed a greater number of *Clostridium* species in the faecal samples of autistic children [79–82]. These data suggest the involvement of *Clostridia* in ASD pathogenesis, as the oral administration of vancomycin to children with ASD led to a regression of the typical symptoms. Because this antibiotic is not absorbed in the gastrointestinal tract and its spectrum of action covers Gram-positive bacteria such as *Clostridium*, it is possible that these microorganisms play a role in ASD development, especially as vancomycin discontinuation led to a reversion of ASD symptoms [83].

The composition of the microbiota has also been explored in children with relapsing-remitting MS [84]. Interestingly, a shorter time to relapse was significantly associated with a reduction in *Fusobacteria* (p = 0.001) an increase in *Firmicutes* (p = 0.003) and the presence of *Archaea euryarchaeota* (p = 0.037). After covariate adjustment, only the depletion of *Fusobacteria* remained significantly associated with relapse risk. These data require deeper investigation but underline a possible connection between disruption of the gut microbiota and MS relapse risk, thus identifying a potential new target for treatment.

### Potential effects of probiotics in neurologic diseases

To date, there are neither guidelines nor clear indications for the use of probiotics to prevent or treat paediatric neurologic diseases. The current evidence on this subject is poor and partial; most of the studies are based on pre-clinical research in animals, such as GF mice subjected to early-life gut modulation of the microbiota or exposed to specific probiotics [85–96], while only a few concern adult subjects [97–106], and even fewer involve children [107–110].

#### Pre-clinical studies

Although the role of probiotics in neurologic disorders is a topic of recent interest, there are many pre-clinical studies in the literature (Table 1). Overall, these studies show potential effects of probiotics in treating neurologic diseases and describe the neural, immunological and metabolic pathways involved [107]. The mechanisms of action are still speculated and unclear, and the choice of strain, dose and timing appears arbitrary.

**Table 1:** Main neurologic effects of probiotics in pre-clinical studies

Authors	Probiotic strains studied	Main neurologic results
Lavasani et al. [86]	*L. paracasei* DSM 13434, *L. plantarum* DSM 15312, *L. plantarum* DSM 15313	Suppression of progression of experimental autoimmune encephalomyelitis and reversion of established disease
Kwon et al. [87]	*S. thermophilus, L. reuteri, B. bifidum, L. acidophilus* and *L. casei*	Suppression of the incidence and progression of experimental autoimmune encephalomyelitis
Chae et al. [88]	*S. thermophilus, L. reuteri, B. bifidum, L. acidophilus* and *L. casei*	Suppression of experimental autoimmune myasthenia gravis
Sun et al. [89]	*C. butyricum*	Neuroprotective properties
Savignac et al. [92]	*B. longum* 1714	Positive impact on cognition
Desbonnet et al. [93]	*B. infantis*	Increase in serotoninergic precursors
Ushakova et al. [86]	*Lactobacilli*	Decrease in astrocyte reaction and motor behaviours
Hsiao et al. [94]	*B. fragilis*	Improvement in anxiety-like, stereotyped, sensorimotor and communicative behaviours

Lavasani et al. reported that a five-day course of 3 *L*actobacillus strains (*L. paracasei* DSM 13434, *L. plantarum* DSM 15312, *L. plantarum* DSM 15313) in mice developing EAE suppressed disease progression and reversed the established disease by down-regulating MOG-reactive T-cells and shifting the dominant immune response from Th1 to Th2; interestingly, beneficial effects were not observed after the administration of a monostrain probiotic; only the three strains taken together yielded therapeutic effects [86].

In a more recent study, Kwon et al. investigated the potential prophylactic and therapeutic effects of IRT5, a combination of *Streptococcus thermophilus*, *Lactobacillus reuteri*, *Bifidobacterium bifidum*, *Lactobacillus acidophilus* and *Lactobacillus casei* (1 × 10^8^ colony forming units [CFU] for each strain) in EAE mice [87]. Pre-treatment with oral administration of IRT5 starting 3 weeks before EAE induction significantly decreased the incidence of EAE compared to controls (roughly 45 vs. 90 %; p < 0.001) and clinical scores (by approximately 50 %; p < 0.001) and reduced infiltration and inflammation in the spinal cord. Furthermore, the use of probiotics in ongoing disease significantly inhibited the progression of EAE and was associated with milder symptoms, although it did not entirely suppress disease progression [87]. The effects of IRT5 have also been examined in murine models of experimental autoimmune myasthenia gravis (EAMG): IRT5 intake starting two weeks before EAMG onset and continuing until six weeks after induction significantly suppressed disease development, with lower clinical scores in treated mice compared to a placebo control group (mean clinical scores 0.93 vs. 1.8; p < 0.05) [88]. Thus, it seems that the prophylactic effects of probiotics are more prominent than the therapeutic ones, likely because of the greater difficulty of modulating already abnormally activated immune cells.

An interesting recent trial demonstrated that a two-week pre-treatment with *Clostridium butyricum* (1 × 10^9^ CFU) in mice had neuroprotective effects against ischaemia/reperfusion injury; in fact, neurologic deficit scores, which were higher in ischaemia/reperfusion mice compared to sham mice (p < 0.01), significantly decreased in treated mice (mean scores 3.5 vs. 2.5; p < 0.05) compared to the ischaemia/reperfusion group, possibly because of anti-oxidant and anti-apoptotic effects [89]. Other studies showed that probiotic administration may lead to anxiolytic effects [90] and improvements in memory and learning [91]. In this regard, *Bifidobacterium longum* 1714 had a positive impact on cognition [92] and *Bifidobacteria infantis* attenuated inflammatory immune responses and elevated serotoninergic precursors, evidence of an antidepressant effect of some strains [93].

Pre-clinical studies have also investigated the potential of chronic administration of probiotics in animal models. One study evaluated the effect of a 6-month treatment with lactic-acid bacteria on the central nervous system of growing rats [85]. Interestingly, after two months, lactic-acid bacteria administration decreased astrocyte reactivity by reducing S-100b and GFAP protein synthesis in the posterior areas of the brain hemisphere and affected the motor behaviour of rats, showing a possible effect in the prevention of neurologic diseases. Beneficial effects on astrocytes seemed to disappear, however, after a 6-month treatment. Thus, the authors recommended supplementing the traditional treatment of neurologic diseases with *Lactobacillus* for only 2–4 months, speculating that prolonged consumption may not be effective because of adaptation in the immunological, gastrointestinal and nervous systems. These data were, however, obtained from experimental models, and the time to adaption to a probiotic treatment may be different in adults and children.

One of the most interesting studies of probiotics in paediatrics was performed by Hsiao et al., who investigated the effect of the oral administration of *Bacteroides fragilis* (1 × 10^9^ CFU) in the maternal immune-activation model of ASD in mouse offspring [94]. They found that probiotic treatment improved anxiety-like, stereotyped, sensorimotor and communicative behaviours, suggesting that microbial modulating therapies may be an effective and safe treatment for ASD. Remarkably, these effects were not related only to *B. fragilis*, as *Bacteroides thetaiotaomicron* administration also significantly improved abnormal behaviours, whereas *Enterococcus faecalis* had no effects, thus suggesting that some specificity is required in bacterial treatment.

Interestingly, a recent systematic review that included 25 randomized, controlled trials performed in animals and 15 in humans showed that *Bifidobacterium* (i.e., *B. longum, B. breve*, and *B. infantis*) and *Lactobacillus* (i.e., *L. helveticus*, and *L. rhamnosus*), with doses between 10^9^ and 10^10^ CFU for 2 weeks in animals and 4 weeks in humans [95]. These probiotics showed efficacy in improving psychiatric disorder-related behaviors including anxiety, depression, ASD, obsessive–compulsive disorder, and memory abilities, including spatial and non-spatial memory.

In addition, Callaghan et al. administered an early-life stressor (i.e., maternal separation) to infant male rats, and investigated the effects of this stressor on conditioned aversive reactions in the rats’ subsequent infant male offspring [96]. They demonstrated, for the first time, longer-lasting aversive associations and greater relapse after extinction in the offspring of rats exposed to maternal separation (F1 generation), compared with the offspring of rats not exposed to maternal separation (F0 generation). These generational effects were reversed by probiotic supplementation, which was effective as both an active treatment when administered to infant F1 rats and as a prophylactic when administered to F0 fathers before conception (i.e., in fathers’ infancy). These findings have high clinical relevance in the identification of early-emerging putative risk phenotypes across generations and of potential therapies to ameliorate such generational effects.

#### Trials on human adults

Clinical trials performed on adults appear to confirm the results achieved in animal studies, suggesting a potential role for probiotics in the treatment of several neurologic diseases (Table 2). Specific probiotics seem to have positive effects on human brain activity, including in healthy subjects. In fact, in a double-blind, placebo-controlled, randomized trial on healthy volunteers, the oral administration of *Lactobacillus helveticus* R0052 and *Bifidobacterium longum* R0175 for 30 days significantly reduced psychological distress and urinary free cortisol level (p < 0.05) [97]. Likewise, in a randomized controlled clinical trial on healthy women, the intake of fermented milk containing *Bifidobacterium animalis* subsp. *lactis, Streptococcus thermophiles, Lactobacillus bulgaricus* and *Lactococcus lactis* subsp. *lactis* for 4 weeks affected responsiveness to negative emotional stimuli by reducing the reactivity of a widely distributed network of brain areas to an emotional attention task (49 % cross-block covariance¸ p < 0.004) containing affective, viscerosensory, and somatosensory cortices [98]. These results demonstrated that the fermented milk affected activity of brain regions that control central processing of emotion and sensation.

**Table 2:** Main clinical trials performed involving adults on the effects of probiotics on neurologic diseases

Authors	Study design	Main neurologic results
Messaoudi et al. [97]	Double-blind, placebo-controlled, randomized trial on healthy volunteers treated with the oral administration of *L. helveticus* R0052 and *B. longum* R0175 vs. placebo for 30 days	Reduced psychological distress and urinary free-cortisol level in probiotics arm
Tillisch et al. [98]	Randomized controlled clinical trial on healthy women treated with fermented milk containing *B. animalis* subsp. *lactis, S. thermophiles, L. bulgaricus* and *L. lactis* subsp. *lactis* vs placebo for 4 weeks	Reduction in responsiveness to negative emotional stimulations in probiotics arm
Matsuzaki et al. [99]	Prospective, uncontrolled treatment with *L. casei* strain Shirota for 4 weeks in patients suffering HTLV-1 associated myelopathy/tropical spastic paraparesis	Improvement in motor dysfunction, urinary symptoms and spasticity in lower extremities
Rao et al. [100]	Double-blind, placebo-controlled, randomized trial in patients with chronic fatigue syndrome treated with *L. casei* strain Shirota vs placebo for 2 months	Decrease in anxiety symptoms in probiotic arm

Interesting data have also been achieved from patients with neurologic disease. The oral administration of *Lactobacillus casei strain Shirota* for 4 weeks in patients suffering HTLV-1 associated myelopathy/tropical spastic paraparesis showed a trend towards improved motor function (p = 0.157) and significantly decreased urinary symptoms (p = 0.0085) and spasticity in the lower extremities (p = 0.015), likely because of an important increase in NK cell activity [99]. Moreover, a two-month course of the same probiotic strain in patients with chronic fatigue syndrome led to a significant decrease in anxiety symptoms compared to controls (p = 0.01) [100].

Positive results were observed also in irritable bowel syndrome in a retrospective study based on the survey of patients records. Trimebutin or mebeverin + anxiolitics + probiotics were associated with the best impact on clinical symptoms mainly in patients with depression [101]. A recent review reported that probiotics appeared clinically and therapeutically relevant to a range of disorders, including chronic fatigue syndrome, fibromyalgia, and restless legs syndrome [102].

On the contrary, the role of probiotic therapy in hepatic encephalopathy is controversial. A meta-analysis of fourteen randomized controlled trials reported a significant improvement in minimal hepatic encephalopathy (odds ratio [OR] 3.91; confidence interval [CI] 2.25–6.80; p < 0.0001), decreased hospitalization rates (OR 0.53; CI 0.33–0.86; p = 0.01) and decreased progression to overt hepatic encephalopathy (OR 0.40; CI 0.26–0.60; p < 0.0001) when probiotics were compared to no treatment/placebo [103]. However, a Cochrane review did not find sufficient evidence to recommend the routine use of probiotic therapy in such patients because of a high risk of systematic and random errors in the trials, which overall showed a reduction in ammonia concentration after probiotic administration but no unequivocal effects on clinically relevant outcomes [104].

#### Trials on children

So far, only a few clinical studies about the use of probiotic strains for paediatric neurologic diseases have been published, and the majority involved children with ASD (Table 3). All studies share many limitations related to design, sample size, inclusion criteria and evaluation of clinical scores. Moreover, none was specifically performed to determine differences in neurologic outcomes or recognize adverse effects of probiotic supplementation [105].

**Table 3:** Main clinical trials performed involving children on the effects of probiotics on neurologic diseases

Authors	Study design	Main neurologic results
Parracho et al. [108]	Randomized, double-blind, placebo controlled study in children with ASD 3–16 years old treated with *L. plantarum* WCFS1 vs placebo for 3 weeks	Improvement of disruptive antisocial behaviours, anxiety and communication problems in probiotic arm
Kaluzna-Czaplinska et al. [109]	Cohort study of children with ASD 4–10 years old treated with *L. acidophilus* strain Rosell-11 for 2 months	Improvement in their ability to concentrate and fulfil orders, with no impact on behavioural responses to other people’s emotions or eye contact
Partty et al. [110]	Randomized trial on infants followed for 13 years, giving *L. rhamnosus* GG vs placebo for the first 6 months of life	At the age of 13 years, 6 out of 35 (17.1 %) children in the placebo group were diagnosed with ASD or attention-deficit/hyperactivity disorder, but none in the probiotic group were
Romeo et al. [111]	Randomized trial in preterm infants treated with *L. reuteri* ATCC 55730 or *L. rhamnosus* ATCC 53103 or no supplementation for 6 weeks	Higher incidence of suboptimal neurological scores in the control group than in both the probiotic groups at 1 year of age

*ASD* autism spectrum disorder

According to one case report, probiotic supplementation improved school records and attitude towards food in a 6-year-old child with ASD; moreover, discontinuation of the intervention led to a regression of symptoms, which was reversed with the reintroduction of probiotics [106].

In a randomized, double-blind, placebo-controlled study, *Lactobacillus plantarum* WCFS1 (daily dose, 4.5 × 10^10^ CFU) was administered to children with ASD from 3 to 16 years of age who were divided into two groups: group I received a placebo during the first feeding period (3 weeks) and a probiotic during the second (3 weeks); vice versa for group II [108]. Behavioural effects were assessed through a standardized Development Behaviour Checklist, which showed improvement in disruptive antisocial behaviours, anxiety and communication problems (p < 0.05) in those treated with probiotics [108].

In a cohort study on 22 children (age range 4–10 years) with ASD, oral supplementation with *Lactobacillus acidophilus* strain Rosell-11 for 2 months improved the ability to concentrate and fulfil orders, but did not affect behavioural responses to other people’s emotions or eye contact [109].

A recent interesting study in children at risk of neurodevelopmental disorders is that of Pärtty et al. [110], who performed a randomized trial on 75 infants followed for 13 years, giving *Lactobacillus rhamnosus* GG to 40 subjects (53.3 %) for the first 6 months of life and a placebo to the other 35 (46.7 %). At the age of 13 years, 6 out of 35 (17.1 %) children in the placebo group were diagnosed with ASD or attention-deficit/hyperactivity disorder (ADHD), but none in the probiotic group were (p = 0.008). These results suggest that early administration of *Lactobacillus rhamnosus* GG started four weeks before expected delivery could possibly reduce the risk of developing ADHD or ASD by mechanisms not necessarily related to the composition of the microbiota, as no single constant component of or change in the microbiota was detected in children with neuropsychiatric disorders compared to those without such disorders. These data represent an interesting preliminary achievement in this field, though they are far from conclusive.

The prophylactic effects of probiotic strains on neurologic outcome have been investigated in a prospective study examining their efficacy in preventing *Candida* late-onset sepsis [111]. A total of 249 preterm infants were divided into three groups: one group (n = 83) supplemented with *Lactobacillus reuteri* ATCC 55730 (5 drops per day in an oil formulation, equivalent to 1 × 10^8^ CFU), one (n = 83) with *Lactobacillus rhamnosus* ATCC 53103 (1 capsule per day, equivalent to 6 × 10^9^ CFU) and the other (n = 83) with no supplementation. All infants were treated for 6 weeks. A neurologic assessment was carried out at 1 year of age using the Hammersmith infant neurological examination showed a statistically significant higher incidence of suboptimal scores in the control group (n = 24) than in both probiotic groups (p < 0.05). Thus, probiotics may be effective in protecting infants from neurologic damage due to sepsis by significantly reducing Candida gastrointestinal colonization and reducing invasive infections, which were more frequent in the control group than in the groups treated with *L. reuteri* and *L. rhamnosus* (4 vs. 1) [111].

### Controversies and future challenges for probiotic-based treatment in patients with neurologic diseases

The use of probiotics in the treatment of neurologic diseases as a routine additive therapy raises some questions.

First, as the positive effects on neurologic diseases are divergent and dependent on the probiotic strain, it is important to perform extensive studies to identify the most appropriate single strain or microbial mixture for each specific neurologic disease [107].

Secondly, probiotics, as the current definition states, need to be administered in adequate amounts. The recommended dose and the optimal duration of treatment have not been completely established; pre-clinical and clinical trials in neurologic diseases differ widely in composition, quantity and timing of probiotic administration. Unfortunately, while there are recommendations for probiotic formulation and dosage for the treatment of other clinical conditions [112], no study of ideal formulation has been performed in patients with neurologic disorders.

In addition, research should focus on the identification of a target population for probiotic therapeutic intervention, including the optimal age of the patient and phase of the disease.

There is concern also about the composition, quality and efficacy of commercially available probiotics. Indeed, probiotic formulations contain only a small amount of beneficial bacteria, usually less than 10 strains that are limited in diversity, unlike our highly variable gut microbiota. Additionally, obligate anaerobes are under-represented in available products, though they correspond to an important part of intestinal bacteria [113]. Moreover, a fatality due to contamination of probiotic supplementation highlighted some doubts about the quality of some products [114]. The ability of each strain and commercialized product to remain viable and effective at a specific target site should also be considered.

Furthermore, most of studies on the effects of probiotics are affected by several limitations and/or confounding factors (i.e., small size sample, different therapies, short follow-up, different evaluation of bowel). Moreover, other investigations have displayed that once pathogenic dysbiosis sets in, probiotics have not proven to be effective and other interventions such as engineered bacteria could be considered [115].

Finally, an extremely important issue involves the tolerability and safety of probiotics in children with neurologic conditions. There is considerable evidence on the safety of probiotics especially related to the use of lactobacilli and bifidobacteria [116]. Although several studies demonstrated the safety of probiotics, some authors have recently called for more research with more clinical trials to identify adverse effects [117]. Two new cases of *Lactobacillus* sepsis have been reported in newborns [118] and, although they are rare, previous cases have also been identified. One occurred in a 6-year-old child with cerebral palsy, mental retardation, and a seizure disorder [119, 120]. While neurologic diseases are not among the known conditions associated with increased risk of probiotic-induced sepsis, we cannot exclude the possibility that patients with severe neurologic conditions may be at greater risk of adverse effects.

## Conclusions

The potential usefulness of probiotics in preventing or treating neurologic diseases is becoming a topic of great interest. However, a better understanding of their mechanisms of action is required to attribute to them a role in improving neurologic manifestations or decreasing the incidence of neurodevelopmental disorders. So far, most of the research on this topic has focused on the mechanisms of the microbiota-gut-brain axis and the best results were observed in children with ASD. A deeper investigation into the efficacy of probiotics in modulating these connections will help clarify the aetiopathogenesis of ASD and some other neurologic diseases and identify new possible targets on which to intervene. Pre-clinical studies suggest promising results, though these findings are not systematic and univocal, but human studies are still limited and not yet conclusive. Well-designed, randomized, controlled clinical trials are needed to confirm experimental results and to identify the appropriate strain, dose and timing for probiotic intervention. Then, a deeper evaluation of safety and tolerability should be carried out in additional studies. However, further clinical research must be encouraged because of the potential impact on neurologic diseases, which are serious public health problems for which there is still no definitive therapy.

## Abbreviations

ASD: autism spectrum disorder
CFU: colony forming units
CI: confidence interval
EAE: experimental autoimmune encephalomyelitis
EAMG: experimental autoimmune myasthenia gravis
GABA: γ-aminobutyric acid
GF: germ free
MS: multiple sclerosis
OR: odds ratio
PD: Parkinson’s disease
